# Riboflavin Biosynthesis and Overproduction by a Derivative of the Human Gut Commensal *Bifidobacterium longum* subsp. *infantis* ATCC 15697

**DOI:** 10.3389/fmicb.2020.573335

**Published:** 2020-09-15

**Authors:** Ana Solopova, Francesca Bottacini, Elena Venturi degli Esposti, Alberto Amaretti, Stefano Raimondi, Maddalena Rossi, Douwe van Sinderen

**Affiliations:** ^1^APC Microbiome Ireland, University College Cork, Cork, Ireland; ^2^Department of Chemistry, University of Modena and Reggio Emilia, Modena, Italy; ^3^BIOGEST-SITEIA, University of Modena and Reggio Emilia, Modena, Italy; ^4^School of Microbiology, University College Cork, Cork, Ireland

**Keywords:** probiotic, vitamin B_2_, gut commensal, vitamin biosynthesis, health benefit

## Abstract

Riboflavin or vitamin B_2_ is the precursor of the essential coenzymes flavin mononucleotide (FMN) and flavin adenine dinucleotide (FAD). Despite increased interest in microbial synthesis of this water-soluble vitamin, the metabolic pathway for riboflavin biosynthesis has been characterized in just a handful of bacteria. Here, comparative genome analysis identified the genes involved in the *de novo* biosynthetic pathway of riboflavin in certain bifidobacterial species, including the human gut commensal *Bifidobacterium longum* subsp. *infantis* (*B. infantis*) ATCC 15697. Using comparative genomics and phylogenomic analysis, we investigated the evolutionary acquisition route of the riboflavin biosynthesis or *rib* gene cluster in *Bifidobacterium* and the distribution of riboflavin biosynthesis-associated genes across the genus. Using *B. infantis* ATCC 15697 as model organism for this pathway, we isolated spontaneous riboflavin overproducers, which had lost transcriptional regulation of the genes required for riboflavin biosynthesis. Among them, one mutant was shown to allow riboflavin release into the medium to a concentration of 60.8 ng mL^–1^. This mutant increased vitamin B_2_ concentration in a fecal fermentation system, thus providing promising data for application of this isolate as a functional food ingredient.

## Introduction

Riboflavin (i.e., vitamin B_2_) is a precursor of flavin mononucleotide (FMN) and flavin adenine dinucleotide (FAD), cofactors acting as essential electron carriers in redox reactions of cell metabolism (LeBlanc et al., 2011). Besides playing an important role in a range of biochemical reactions, riboflavin serves as a signaling molecule in bacterial quorum sensing and bacterium-plant interactions, while intermediates of the riboflavin biosynthesis pathway are also involved in host-microbe signaling (Rajamani et al., 2008; Corbett et al., 2014; Dakora et al., 2015). Only plants, fungi, and various eubacteria encode the complete enzymatic machinery for *de novo* riboflavin biosynthesis (Bacher et al., 2000; Fischer et al., 2004). Humans need to regularly obtain this vitamin through their diet as they are unable to synthesize or store it. For this reason, riboflavin is an ingredient in various fortified foods and multivitamin supplements. Absorption of riboflavin occurs in the small and large intestine through specific carriers (Thakur et al., 2017). The recommended daily allowance for riboflavin increases with age, reaching up to 1.1 and 1.3 mg in adult women and men, respectively, and is the highest (1.6 mg) in lactating mothers (Institute of Medicine, 1998).

Riboflavin had been produced by chemical means until commercially competitive microbial processes were developed to facilitate large-scale production. Biotechnological processes with various microbial cell factories such as *Bacillus subtilis*, *Candida flareri*, and especially, *Ashbya gossypii*, represent typical examples of white biotechnology (Stahmann et al., 2000; Ledesma-Amaro et al., 2015). In recent years, vitamin production by lactic acid bacteria (LAB) and bifidobacteria has received increased attention. These organisms are widely exploited by the food and pharmaceutical industries as starters for fermented products and/or as probiotic supplements aiming to improve human health. Utilization of vitamin-producing bacteria in food manufacturing has the obvious advantage of achieving *in situ* food fortification, which represents a non-artificial and economically viable strategy (Thakur et al., 2016). Vitamin-producing probiotic strains that may colonize, at least transiently, the human intestine, have the advantage of providing the host with a continuous supply of this micronutrient. Certain members of the genus *Bifidobacterium* have previously been proposed to supply vitamins, but, unlike folate, riboflavin-producing bifidobacteria have so far not been assessed (Pompei et al., 2007a,b; D’Aimmo et al., 2012; Sugahara et al., 2015). Notably, among bifidobacteria the presence of a complete riboflavin biosynthesis pathway and associated cluster has so far only been reported in *B. longum* subsp. *infantis* (Sela et al., 2008; Kwak et al., 2016).

**FIGURE 1:**
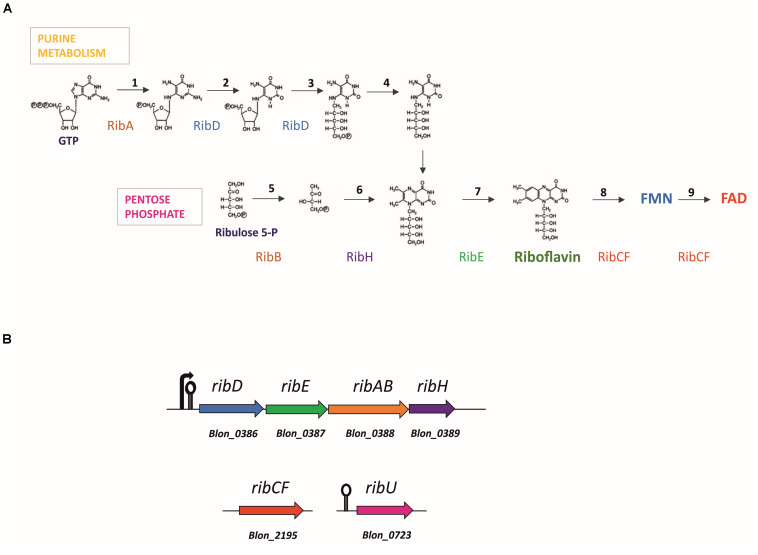
Flavin adenine dinucleotide (FAD) biosynthesis and corresponding gene loci predicted in the genome of *Bifidobacterium longum*. **(A)** General bacterial pathway of FAD biosynthesis. Stoichiometrically, the formation of riboflavin requires one equivalent of GTP and two equivalents of ribulose 5-phosphate. Reactions are catalyzed by enzymes: RibAB (1, 5), bifunctional GTP cyclohydrolase II [EC:3.5.4.25]/(3,4-dihydroxy-2-butanone 4-phosphate synthase [EC:4.1.99.12]; RibD (2, 3), bifunctional diaminohydroxyphosphoribosylaminopyrimidine deaminase [EC:3.5.4.26]/5-amino-6-(5-phosphoribosylamino)uracil reductase [EC:1.1.1.193]; unknown enzyme (4), 5-amino-6-(5-phospho-D-ribitylamino)uracil phosphatase [EC:3.1.3.104]; RibH (6), 6,7-dimethyl-8-ribityllumazine synthase [EC:2.5.1.78]; RibE (7) riboflavin synthase [EC:2.5.1.9]; RibCF (8, 9), bifunctional riboflavin kinase [EC:2.7.1.26]/FAD synthetase [EC:2.7.7.2]; Based on Bacher et al., 2000; **(B)** Gene loci encoding for the riboflavin synthesis enzymes in *Bifidobacterium infantis* ATCC 15697. Arrow marks the promoter, lollipop marks the FMN riboswitch upstream the *rib* cluster and *ribU* gene.

Bacteria synthesize FMN and FAD according to the pathway outlined in Figure 1, starting from one molecule of guanosine-5′-triphosphate (GTP) and two molecules of ribulose-5-phosphate (Vitreschak et al., 2002; Fischer et al., 2004). The RibAB, RibD, RibE and RibH riboflavin biosynthetic enzymes are encoded by the *rib* operon, while the genes encoding RibCF and RibU, the latter a transporter for the uptake of pre-formed riboflavin, are typically located elsewhere on the chromosome (Vitreschak et al., 2002; Burgess et al., 2006a; Thakur et al., 2016). The first step of riboflavin biosynthesis is catalyzed by the enzyme GTP cyclohydrolase/dihydroxy-2-butanone-phosphate synthase (RibAB), which is responsible for the formation of formate and 3,4-dihydroxy-2-butanone 4-phosphate (DHBP) from D-ribulose 5-phosphate and 2,5-diamino-6-beta-ribosyl-4(3H)-pyrimidinone 5’-phosphate (DARP) from GTP, which are precursors of the aromatic ring of riboflavin. A reductase/deaminase (RibD) catalyzes the subsequent reduction of the ribose side chain in DARP to produce 5-amino-6-(5-phospho-D-ribitylamino)uracil. The last two steps of the pathway are then completed by a lumazine synthase (RibH) and a riboflavin synthase (RibE). The obtained riboflavin is then converted by phosphorylation into FMN and FAD by a reaction catalyzed by a riboflavin kinase/FAD synthase (RibCF; Figures 1A,B). In Firmicutes and in Actinobacteria, *de novo* biosynthesis is regulated through a feedback mechanism involving transcriptional attenuation via an FMN-sensing mRNA riboswitch (Mironov et al., 2002; Vitreschak et al., 2002). If present, FMN is bound with high affinity by the 5’-untranslated region (5’-UTR) of the nascent transcript of the *rib* operon, which serves as the FMN-binding aptamer. The binding causes the transcript to switch to its anti-antiterminator structure, allowing a termination hairpin to form thereby prematurely ending *rib* operon transcription (Kil et al., 1992; Mironov et al., 2002; Vitreschak et al., 2002).

Roseoflavin is an antibacterial compound and a structural analog of riboflavin, produced by *Streptomyces davawensis* (Mack et al., 1998; Ott et al., 2009). This antimetabolite binds to the active site of FAD synthetase and flavokinase yielding inactive complexes of roseoflavin mononucleotide (RoFMN) and roseoflavin adenine dinucleotide (RoFAD), thereby inhibiting major cellular reactions. Additionally, RoFMN mimics FMN in the riboswitch regulation and suppresses *de novo* vitamin production (Mack et al., 1998; Ott et al., 2009; Ludwig et al., 2018). Isolation of spontaneous roseoflavin-resistant mutants is a reliable method to obtain riboflavin-overproducing strains of various species, since increased vitamin production counteracts roseoflavin toxicity by competitive binding to FAD synthetase (Matsui et al., 1982). This approach, which does not involve deliberate genetic engineering and may thus be acceptable for food applications, has generated various riboflavin-producing mutants that can be exploited to produce riboflavin-enriched foods employing LAB and propionibacteria (e.g., *Lactococcus lactis*, *Lactobacillus plantarum*, *Lactobacillus fermentum*, *Propionibacterium freudenreichii*; Burgess et al., 2004, 2006b; LeBlanc et al., 2005, 2006; Capozzi et al., 2011; Russo et al., 2014; Juarez Del Valle et al., 2016; Mohedano et al., 2019; Yepez et al., 2019; Ge et al., 2020).

The biosynthetic pathway for riboflavin had previously been predicted for some bifidobacterial strains, such as the type strain *Bifidobacterium longum* subsp. *infantis* ATCC 15697 (hereinafter referred to as *B. infantis* ATCC 15697; Kwak et al., 2016). However, riboflavin-(over)producing strains have never been described and little is known about biosynthesis and regulation of this vitamin in bifidobacteria. In the present study, the presence of the riboflavin and FMN/FAD biosynthetic pathway was assessed in all annotated *Bifidobacterium* genomes. Spontaneous roseoflavin-resistant mutants of *B. infantis* ATCC 15697 were obtained and were shown to constitutively produce riboflavin, which was released in the growth medium. Promising evidence for or a potential application in the probiotic industry was obtained in a fecal fermentation system, in which the strain was shown to increase the overall riboflavin concentration.

## Materials and Methods

### Comparative Bioinformatic Analysis

Protein coding sequences derived from a total of 662 representative strains from 83 bifidobacterial (sub)species were retrieved from the NCBI Reference Sequence collection Refseq^1^ database and constituted the input for the comparative analysis. The occurrence and distribution of the riboflavin biosynthesis genes Blon_386–389 and Blon_2195 across *Bifidobacterium* was performed using BLASTP alignments (Altschul et al., 1990) against a database build from the open reading frames (ORFs) collection of each bifidobacterial species. Proteins homologous to any of the riboflavin-associated gene products from *B. infantis* across the *Bifidobacterium* genus were identified using cut-off values of 40% of similarity across 70% of protein length and an *e*-value of < 0.0001 for significance. The result of the alignments was represented as a heatmap using the “heatmaply” package implemented in the statistical software R^2^ and using a two-way hierarchical clustering with a color gradient expressing the degree of sequence similarity across bifidobacterial species, which were also classified by the origin of isolation. A homologous cluster was defined as present across bifidobacterial genomes when the four genes constituting the *ribDEABH* cluster were found co-located within the same genomic region with an average similarity above 40% at protein level.

Prediction of the FMN riboswitch in *B. infantis* and *Bifidobacterium* was performed using Infernal tool v1.1.3 implemented in Bioconda environment^3^ and the alignment of the FMN riboswitch was performed using Muscle alignment tool v3.8.31 (Edgar, 2004) and visualized using https://alignmentviewer.org/.

### Chemicals and Media

All chemicals were purchased from (Sigma-Aldrich, St. Louis MO, United States) unless otherwise stated.

Bifidobacteria were routinely cultured anaerobically in Lactobacilli de Man, Rogosa and Sharpe (MRS) Broth (BD Difco, Sparks, MD, United States) containing 0.5 g L^–1^ cysteine HCl.

Riboflavin production in pure *Bifidobacterium* cultures was tested in riboflavin-free semi-synthetic medium mSM7. Based on SM7 medium (Pompei et al., 2007a) with modifications, mSM7 is composed assembling three sterile solutions: (A) 10 × carbohydrate solution (sterilized by autoclaving at 121°C for 20 min); (B) 10 × vitamin solution (pyridoxine, 2 mg L^–1^; nicotinic acid, 2 mg L^–1^; thiamine, 2 mg L^–1^; calcium pantothenate, 1 mg L^–1^; p-aminobenzoic acid, 0.05 mg L^–1^; biotin, 0.05 mg L^–1^; ascorbic acid, 1 mg L^–1^; adenine sulfate, 20 mg L^–1^; xanthine, 40 mg L^–1^; vitamin B_12_, 0.7 mg L^–1^; folate, 0.5 mg L^–1^; sterilized by filtration); (C) 1.25 × basal solution (Difco^TM^ Casamino Acids, vitamin assay BD, Franklin Lakes, NJ, United States), 10 g L^–1^; sodium acetate, 10 g L^–1^; (NH_4_)_2_SO_4_, 5 g L^–1^; urea, 2 g L^–1^; MgSO_4_⋅7 H_2_O, 0.2 g L^–1^; FeSO_4_⋅7 H_2_O, 0.01 g L^–1^; MnSO_4_⋅7 H_2_O, 0.007 g L^–1^; NaCl, 0.01 g L^–1^; Tween 80, 1 g L^–1^; cysteine, 0.5 g L^–1^_;_ autoclaved for 30 min at 110°C after adjusting the pH to 7.0. Batch fermentations were carried out in mSM7 supplemented with glucose, lactose, fructo-oligosaccharides (FOS) [P95, degree of polymerization (DP) 2–8, Beneo-Orafti, Mannheim, Germany], galacto-oligosaccharides (GOS, DP 3–9, Vivinal, FrieslandCampina, Amersfoort, Netherlands), xylooligosaccharides (XOS, DP 2–6, FrieslandCampina), or inulin (HP, DP > 23, Beneo-Orafti), to achieve the final concentration of 10 g L^–1^.

### Isolation of Roseoflavin-Resistant Mutants

The approach taken to isolate roseoflavin resistant mutants is summarized in Supplementary Figure S1. *B. infantis* ATCC 15697 was cultured overnight in 5 mL of mMRSlac [i.e., modified De Man et al. (1960) and Sharpe medium made from first principles and supplemented with 1% lactose instead of glucose]. Cells were collected by centrifugation (6,000 × *g* for 10 min), washed twice with 5 mL of sterile H_2_O, and resuspended in 10 mL of mSM7lac (1% lactose). The culture was allowed to grow for 5 h under anaerobic conditions. Aliquots of 100 μL of cell suspension were spread onto agar plates of mSM7lac supplemented with 0, 25, or 50 μg mL^–1^ roseoflavin and incubated anaerobically at 37°C for 48 h. Following isolation of roseoflavin-resistant derivatives, the promoter and the 5’-UTR regions of the *rib* operon of a number of such derivatives were PCR amplified (using primers 5’ TTCTCCGATACGGGCGATTG 3’ and 5’ TTCGTGGCATCGACCGACAG 3’) and the obtained products were subjected to Sanger sequencing (Eurofins Genomics, Germany). The obtained sequences were aligned using Muscle (Edgar, 2004) and the alignment visualized using Alignmentviewer^4^.

### Cultivation Conditions

The wild-type (WT) of the type strain *B. infantis* ATCC 15697 and selected roseoflavin-resistant derivatives were subcultured in mSM7 supplemented with glucose (mSM7glucose) and were then incubated anaerobically at 37°C for 48 h. Growth and production of intracellular and extracellular riboflavin were assayed in liquid cultures of mSM7 supplemented with glucose, lactose, FOS, GOS, XOS, or inulin. Cultures were propagated three times in the same medium before measuring riboflavin concentration in the supernatants or in the cell extracts. Growth was determined by measuring the final optical density at 600 nm (OD_600_).

Controlled-pH batch cultivation was carried out in triplicate in laboratory-scale bioreactors (500 mL Mini Bio, Applikon Biotechnology, Delft, the Netherlands) containing 0.3 L of mSM7 supplemented with lactose (mSM7lac). Bioreactor was inoculated (10% v/v) with exponential phase pre-cultures grown in the same medium. The culture was kept at 37°C under CO_2_ atmosphere and stirred at 250 rpm. The pH was continuously measured (Mettler Toledo InPro 3030/325) and kept at 5.5, the optimal pH value for bifidobacteria (Amaretti et al., 2007), by automatic titration with 1 M NaOH. Samples were periodically collected for analysis of carbohydrates, fermentation products, and growth.

Carbohydrates and fermentation products were analyzed in the supernatants using an HPLC apparatus with refractive index detector (1200 System, Agilent Technologies, Waldbronn, Germany). Elution was carried out with 0.6 mL/min of 0.005 M H_2_SO_4_ through an ion exclusion column (Aminex HPX-87 H, Bio-Rad Laboratories, Inc., Hercules, CA, United States) maintained at 60°C.

### Riboflavin Assay

Riboflavin concentration was assayed in cell extracts and culture supernatants. 50 mL of the culture was centrifuged at 9.000 × *g* for 10 min at 4°C. The supernatant was filtered through a 0.22 μm filter and frozen at -20°C. The biomass was washed with the same volume of 0.1 M Na-phosphate buffer pH 7.0. The pellet was resuspended in the buffer (1/10 of the initial volume of the culture), and the suspension was frozen at -20°C. The thawed biomass was passed through the One Shot Cell Disrupter (Constant Systems, Ltd., Daventry, United Kingdom) at 40 KPsi. The cell extract, obtained by centrifugation of the sample at 13.000 × *g*, 15 min, 4°C was maintained at -20°C.

Riboflavin concentration was quantified using a microbiological bioassay with *Lactobacillus casei* subsp. *rhamnosus* ATCC 7469 as test organism, based on Woodson et al. (1946). Growth of the test organism was measured in 5 mL of Riboflavin Assay Medium (BD, Flanklin Lakes, NJ, United States) supplemented with 5 mL of properly diluted sample and compared with a calibration curve in the range of 0–300 ng mL^–1^, according to the protocol described by the medium manufacturer. Microbiological assay measurements were replicated at least six times.

### Real-Time Quantitative qRT-PCR

Differential expression of *rib* genes was confirmed by real-time quantitative RT-PCR (qRT-PCR). *B. longum* subsp. *infantis* ATCC 15697 (WT) and its spontaneous mutant ROS25 were grown in mSM7lac supplemented or not with 20 ng mL^–1^ riboflavin until mid-exponential growth phase (OD_600nm_ 0.45–0.7). Cells were harvested by centrifugation at 6.000 × *g* for 10 min at 4°C and the pellet was immediately frozen at -80°C. For RNA isolation, cells were resuspended in 0.5 mL of TE buffer (10 mM Tris-HCl, 1 mM EDTA, pH 8.0), transferred to a 2 mL screw cap tube. 0.5 g of glass beads (∼100 μm in diameter), 50 μL of 10 % sodium dodecyl sulfate (SDS; Sigma-Aldrich, Saint Louis, MO, United States), and 500 μL of premixed phenol:chloroform:isoamyl alcohol (25:24:1) were added to the thawed cells in the screw cap tube. Cells were disrupted using three cycles of 60 s of bead-beating with a 1 min interval on ice. The cell lysate was cleared by 10 min centrifugation at 10.000 × *g*, at 4°C. The upper phase was extracted with 500 μL of chloroform:isoamyl alcohol (24:1). The two phases were separated by centrifugation (10 min, 10.000 × *g*, 4°C) and total RNA was isolated from the aqueous phase using the High Pure RNA Isolation Kit (Roche Molecular Systems, Inc., Pleasanton, CA, United States), according to the manufacturer’s instructions. Total RNA was treated with DNase I RNAse-free (Roche Molecular Systems, Inc.) according to manufacturer’s instructions. cDNA synthesis was performed using total RNA as a template and Transcriptor Reverse Transcription Kit (Roche Molecular Systems, Inc.) according to manufacturer’s instructions. qRT-PCR experiments were carried out using SYBR Green MasterMix (Thermo Fisher Scientific, Waltham, MA, United States) using cDNA samples as a template. Primers to amplify *ribD* and two housekeeping genes were designed using the Universal ProbeLibrary Assay Design Center (Roche Molecular Systems, Inc.). The *groEL* and *gyrA* genes were used as housekeeping genes with a presumed constitutive level of transcription to correct for variability in the initial amount of total RNA. All qRT-PCRs were performed in triplicate by means of a LightCycler 480 system (Roche Molecular Systems, Inc.) instrument using 384-well plates. Thermal cycling conditions were as recommended by the manufacturer (Roche Molecular Systems, Inc.). The 2^–ΔΔ^*^CT^* method was used to calculate relative changes in gene transcription determined from qRT-PCR experiments. Relative transcription levels of targeted genes from WT and ROS25 were compared using the Student’s *t*-test and were considered significantly upregulated when a *p*-value of < 0.05 was obtained.

### RNA Isolation and Sequencing

*B. longum* subsp. *infantis* ATCC 15697 (WT) and its spontaneous mutant ROS25 were grown in mSM7lac until mid-exponential growth phase (OD_600nm_ of 0.7, to obtain a sufficient RNA yield) for RNA-seq experiments performed in duplicate. Cells were then harvested by centrifugation and the obtained pellets were frozen and stored at -80°C. For RNA extraction, total RNA of each of the cultures was mixed with 800 μl of QIAzoL Lysis Reagent (Qiagen, Venlo, Netherlands) in a sterile tube containing glass beads (Merck, Darmstadt, Germany). Cells were lysed by alternating 2 min of stirring the Precellys 24 homogenizer (Bertin instruments, Montigny-le-Bretonneux, France) with 2 min of static cooling; this step was repeated three times. The sample was centrifuged at 12,000 × *g* for 15 min and the upper phase was recovered. The RNA was purified using the RNAesy Mini Kit (Qiagen), following the manufacturer’s protocol. RNA concentration and purity were evaluated by a Picodrop microliter spectrophotometer (Victory Scientific, Cambridge, United Kingdom). Before RNA sequencing, 2.5 μg of total RNA was treated to remove the ribosomal RNA by the Ribo-Zero Magnetic Kit (Illumina, Inc., San Diego, CA, United States), followed by purification of the rRNA-depleted sample by ethanol precipitation. RNA was processed according to the manufacturer’s protocol. The yield of rRNA depletion was confirmed by a Tape station 2200 (Agilent Technologies, Santa Clara, CA, United States). A whole transcriptome library was constructed using the TruSeq Stranded RNA LT Kit (Illumina, Inc.) and samples were loaded into a NextSeq High Output v2 Kit Chemicals 150 cycles (Illumina), according to the technical support guidelines.

### RNA-Seq Analysis

Following sequencing, the reads were depleted of adapters, quality filtered (with overall quality, quality window and length filters) and aligned to the *B. infantis* ATCC 15697 reference genome (genomic features model GTF file^5^) through bowtie2 aligner^6^. The alignment SAM files were further processed using Samtools to obtain BAM files necessary to obtain matrices with read counts per gene (normalized by gene length). Differential gene expression (DGE) analysis was performed using the R statistical platform and the DESeq2 package available as part of the Bioconductor release (v.3.9). As a pre-processing step, rows with zero counts (unmapped genes) were discarded from the count matrices. Differential expression analysis was performed on the count matrices using the DESeq function in DESeq2. Genes with an FDR-adjusted *p*-value of < 0.05 and a log2-fold change of > 3 were considered significantly upregulated.

### Determination of β-Galactosidase Activity and Protein Concentration in the Supernatant

β-galactosidase activity and total protein concentration were determined in the supernatant of ROS25 and WT cultures grown for 6 h in mSM7lac, obtained from three independent experiments. β-galactosidase activity assay was performed with *o*-nitrophenyl-β-D-galactopyranoside (*o*NPG). The pH of the supernatant was corrected to 7.0 with a NaOH 3 M solution. One mL of the sample was incubated at 37°C for 3 min after the addition of 0.2 mL of 4 mg mL^–1^ *o*NPG. The reaction was stopped by adding 0.5 mL of 1 M Na_2_CO_3_. The absorbance of the sample was read at 420 nm. One unit of β-galactosidase was defined as the amount of enzyme required to release 1 μmol of nitrophenol per minute under the assay conditions. The activity was normalized to the OD_600_ units of the culture.

To quantify protein levels in the supernatant, the culture was centrifuged at 9,000 × *g* for 10 min at 4°C. The volume of supernatant corresponding to two units of OD_600_ of the culture was transferred to a 2 mL tube and was mixed with the same volume of 20% (w/v) trichloroacetic acid. The sample was vortexed, incubated for 1 h in ice, then centrifuged at 13,000 × *g* for 10 min at 0°C. The pellet was washed with 300 μl cold acetone. After gently mixing of the suspension by inversion, the sample was centrifuged at 13,000 × *g* for 10 min at 0°C, and the pellet was allowed to dry for 30 min at room temperature. The sample was resuspended in 20 μl of Laemmli Loading buffer (Bio-Rad Laboratories, Inc., Hercules, CA, United States) and supplemented of 2 μl NaOH 1 M. Proteins were separated by SDS-PAGE using a 5% stacking and 10% resolving gels. Coomassie brilliant blue-stained gels were scanned in transmissive mode using a white light source with the GS-800 Calibrated Densitometer (Bio-Rad Laboratories, Inc.). Taking into account that the proteins of each run derived from the same amount of biomass, the total protein levels of the supernatants were presented as percentage of the highest value obtained in the data set. In the permeability assays, means of WT and ROS25 were compared with Student’s *t*-test and were considered significantly different when a *p*-value of < 0.05 was obtained.

### Fecal Cultures

Fresh fecal samples were obtained from 10 healthy volunteers (five men and five women, aged 25–50 years), who had provided written informed consent according to the experimental protocol with ref. no. 968/2019/SPER/UNIMO-RIBOBIF and approved by the local research ethics committee (Comitato Etico dell’Area Vasta Emilia Nord, Italy). Feces were homogenized with 3 mm sterile borosilicate glass beads in mSM7 medium supplemented with 10 % GOS (mSM7GOS), in an anaerobic cabinet (Concept Plus, Ruskinn Technology, Ltd., Bridgend, United Kingdom), under an 85% N_2_, 10% CO_2_, and 5% H_2_ atmosphere. Analysis of riboflavin content in feces was carried out on dilutions of the fresh 10 % slurry.

Two sets of fermentation experiments were run in parallel: (A) fecal cultures inoculated with the fecal slurry pasteurized for 10 min at 80°C; (B) fecal cultures inoculated with a viable microbiota. Fifty mL of mSM7GOS were supplemented with 5 mL of 10% fecal slurry (pasteurized or not) and with 2.5 mL of ROS25 or WT suspension (2.5 mL of mSM7GOS in the control). The inocula of ROS25 and WT were prepared by centrifuging a 24 h culture in mSM7GOS at 6,000 × *g* for 10 min at 4°C, and resuspending the pellet in 1/10 of the volume of the same medium. Riboflavin content was analyzed after inoculation, and after 16 and 24 h of incubation at 37°C in anaerobic conditions. Two-way analysis of variance (ANOVA), followed by Tukey’s *post hoc* test, was used to evaluate differences between carbon sources and strains (WT and ROS25) in pure culture experiments and between time points and groups (control, WT, an ROS25) in fecal cultures. Differences were considered significant when a *p*-value of < 0.05 was obtained.

## Results

### Identification of Riboflavin Biosynthesis Genes in *Bifidobacterium* Genomes

The riboflavin and FMN/FAD biosynthetic pathway was reconstructed in annotated *Bifidobacterium* genomes available in the Kyoto Encyclopedia of Genes and Genomes (KEGG) database. All genes necessary for *de novo* riboflavin biosynthesis were found in the genome of *B. infantis* ATCC 15697 (Figure 1B), as previously reported by Kwak et al. (2016). The set of genes includes a complete *rib* operon (*ribD*, *ribE*, *ribAB*, and *ribH* homologues, corresponding to locus tags Blon_0386 to Blon_0389) encoding the enzymes for riboflavin biosynthesis from GTP and ribulose-5-phosphate, and an unconnected *ribCF* (Blon_2195) homologue encoding a bifunctional riboflavin kinase/FMN adenylyltransferase, responsible for the conversion of riboflavin into FMN and FAD (Figures 1A,B). The genome also harbors a *ribU* homologue (Blon_0723), predicted to encode a transporter for the uptake of pre-formed riboflavin located at a genomic position that is unconnected to that of the *rib* operon or *ribCF* (Figure 1B).

**FIGURE 2:**
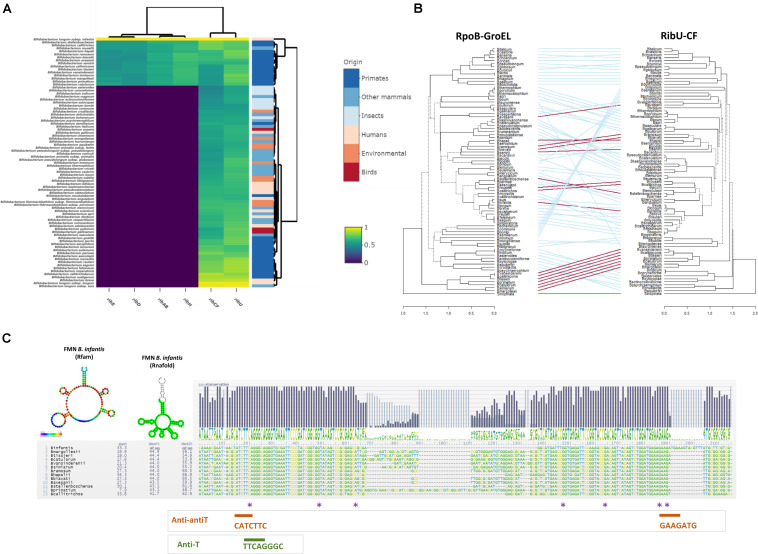
Comparative analysis of riboflavin biosynthesis in *Bifidobacterium*. **(A)** Comparative heatmap representing the distribution of riboflavin-associated genes from *Bifidobacterium infantis* ATCC 15697 across the *Bifidobacterium* genus. Color gradients indicate the percentage of identity in BLASTP alignments of open reading frames (ORFs) derived from bifidobacterial genomes grouped by origin of isolation; **(B)** Entanglement trees obtained from the concatenation of *rpoB, groEL* vs. *ribU, ribCF* genes across the genus *Bifidobacterium*. The two Neighbor Joining (NJ) trees were built using the MEGA package (statistical validation of 100 bootstrap replicates) and visualized using the “Dendextend” package in R v3.6.2. Connecting lines are color coded based on the presence (red) or absence (blue) of a *rib* cluster. The outgroup is indicated in green. **(C)** Sequence alignment of FMN region in *B. infantis* and 13 bifidobacterial strains containing a complete *rib* cluster. Sequence conservation is indicated with a logo for the consensus. Colored asterisks indicate mutations in FMN riboswitch region upstream of the *rib* cluster identified in ROS-resistant isolates. Prediction of the FMN folding was based on Rfam aligment (bases color-coded based on sequence conservation to the Rfam model RF00050) and was predicted by Rnafold. The obtained structures were obtained using the Infernal and Rnafold predictions in https://structrnafinder.integrativebioinformatics.me/ (Arias-Carrasco et al., 2018).

The protein sequences deduced from the *rib* genes of *B. infantis* ATCC 15697 were utilized as query in a search aimed at identifying homologues in an ORF dataset that was extracted from 662 genomes, being representative of 83 bifidobacterial (sub)species. Our combined BLASTP and MCL clustering analysis revealed that *de novo* riboflavin biosynthesis appears to be a rather uncommon feature among members of the genus *Bifidobacterium*, since homologues of the complete *rib* operon were identified in just 16 out of 83 bifidobacterial species, most of which (14 out of 16) had been isolated from primates (Figure 2A). In contrast, homologues of *ribCF* and *ribU* are conserved across all members of the genus, occurring as single copy genes in all investigated species (Figure 2A). This indicates a general ability of bifidobacteria to rely on the provision of pre-formed, extracellular vitamin B_2_ to ensure a constant FAD/FMN supply. Notably, homologues of the *ribCF* and *ribU* genes from *B. infantis* have been identified as essential genes in *Bifidobacterium breve* UCC2003 using a combination of Tn5 transposon library and TraDIS sequencing (Ruiz et al., 2017), supporting their crucial involvement in specific housekeeping functions in the *Bifidobacterium* genus. Inspection of the phylogenetic trend of the two housekeeping genes *rpoB* and *groEL*, and that of the riboflavin-associated genes *ribCF* and *ribU* substantiated that the latter had followed the same evolutionary course of other housekeeping functions of this genus, indicating that the uptake and utilization/conversion of riboflavin represents a core feature of *Bifidobacterium* that had already been incorporated in the genomes of its ancestors (Figure 2B). In support of this notion, homologues of the *ribCF* gene harbored by bifidobacterial species isolated from insects presented the lowest similarity compared to species from other ecological niches (40–50% of identity across 70% of sequence length; Figure 2A). As bifidobacterial isolates from insects have been identified as being most related to the ancestor of the genus *Bifidobacterium* (Milani et al., 2014), the ability to synthesize riboflavin seems a genetic feature that was not originally present in this genus. Instead, this property must have been more recently acquired by certain bifidobacterial species, likely in an ecological context of riboflavin deficiency.

A PSI-BLAST analysis against the *nr* database was performed using the amino acid sequence of the riboflavin synthase (RibE) as a query. Approximately 90 different taxa encoding RibE above 50% identity (across 100% of sequence length) were identified. Phylogenetic inference revealed that the *rib* clusters found in the 15 non-human associated bifidobacteria (Figure 2A) are related and form a separate clade when compared to the *rib* cluster present in *B. infantis* (Supplementary Figure S2). Phylogenetic relationship analysis suggests that *B. infantis* acquired and retained, or lost the primate-associated *rib* cluster and then reacquired a *rib* cluster via a separate horizontal transfer event (Supplementary Figure S2), perhaps facilitated by another intestinal bacterium, which could have been either a gut commensal or pathogen such as *Streptococcus* or *Clostridium*.

In order to verify our findings and test the functionality of the *rib* cluster we focused on the human-gut commensal *B. longum* subsp. *infantis* ATCC 15697, which was predicted to encode the complete *de novo* riboflavin biosynthesis pathway.

### FMN Riboswitch Comparison Across *Bifidobacterium*

To investigate the presence and sequence conservation of the FMN riboswitch, a sequence of 500 bp in the 5’-UTR upstream of *ribD* was extracted from the *Bifidobacterium* spp. genomes bearing the complete *rib* operon. The structural elements responsible for the presumed FMN riboswitch, comprised of the antiterminator (AntiT, 5’ -TTCAGGGC-3’) which promotes *rib* transcription and two anti-antiterminators (Anti-antiT: 5’-CATCTTC-3’ and 5’-GAAGATG-3’) which pair in the presence of FMN/FAD and cause premature transcriptional termination (Winkler et al., 2002), were detected in all 5’-UTRs. Aligning the FMN riboswitch sequences of various bifidobacteria revealed that Anti-T and Anti-antiT are located in a conserved region (Figure 2C), suggesting that the FMN riboswitch is functional and regulates *rib* operon transcription. An FMN riboswitch was also identified in the 5’-untranslated region (5’-UTR) of the *ribU* uptake system, indicating that riboflavin synthesis and uptake are both controlled by the level of flavin cofactors and vitamin in the environment.

#### Riboflavin Production in *B. longum* subsp. *infantis* ATCC 15697 and Characterization of Roseoflavin-Resistant Mutants

To verify the predicted ability of *B. infantis* ATCC 15697 to produce riboflavin, it was cultured in a semi-synthetic medium lacking riboflavin (mSM7lac), allowing growth only if the *de novo* biosynthetic pathway for this vitamin was functional. The strain grew abundantly (final OD_600nm_ = 3.0) and produced the vitamin, albeit at a low level (less than 0.2 ng mL^–1^ in a cell extract after 48 h of incubation), without any detectable release of riboflavin in the medium.

To obtain spontaneous mutants with improved riboflavin production, *B. infantis* ATCC 15697 was exposed to 25 and 50 μg mL^–1^ roseoflavin on mSM7lac plates. Roseoflavin-resistant colonies were obtained at a similar mutational frequency (3.4 × 10^–7^) with either of the two concentrations of this antimetabolite. A selection of seven roseoflavin-resistant isolates, hereinafter identified with a ROS designation (Table 1), was assayed for vitamin B_2_ production. All ROS mutants exhibited an increased riboflavin production level of at least one magnitude when compared with the WT. The concentration of vitamin released in the supernatant by the mutants ranged from 2.5 to 35 ng mL^–1^ (Table 1).

**TABLE 1:** Mutations identified in flavin mononucleotide (FMN) riboswitch of roseoflavin-resistant mutants and extracellular riboflavin.

Mutant	Position in FMN Riboswitch	Base Wild-Type (WT)	Base mutant	Riboflavin concentration (ng mL^–^^1^)
ROS20	6	C	T	18
ROS21	41	C	T	2.5
ROS22	31	G	A	6.5
ROS23	102	G	A	16
ROS25	105	G	A	35
ROS26	81	G	A	4.5
ROS29	64	G	A	8
WT	–	–	–	< 0.2

*Average values are shown (*n* = 3).*

The promoter and the 5’UTR regions of the *rib* operons of the seven assessed ROS mutants were sequenced, to identify the mutations likely responsible for the increase in riboflavin production. The mutants all harbored single point mutations at distinct positions of the putative FMN riboswitch (Figure 2C and Table 1). The identified point mutations were either G → A or C → T transitions. Three mutants acquired mutations in one of the two anti-antiterminator regions (Figure 2C and Table 1). The highest riboflavin production was observed in mutant ROS25, which was shown to carry a G → A transition in the anti-antiterminator region of the riboswitch (Table 1).

### Riboswitch Disruption and Global Gene Expression Changes in Spontaneous Mutant ROS25

**FIGURE 3:**
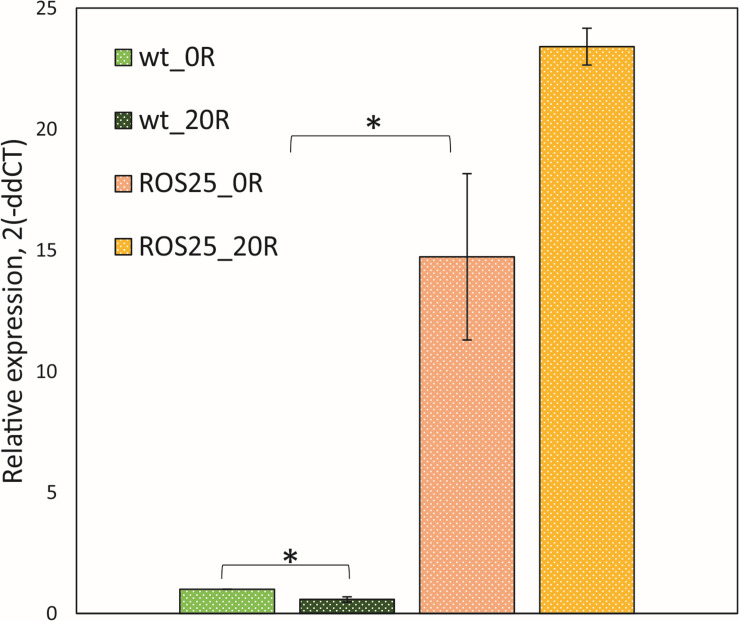
Roseoflavin-resistant isolates. Relative expression of *ribD* in wild-type (WT) and ROS25 cells grown to the mid-exponential growth phase in mSM7lac with 0 or 20 ng mL^–1^ riboflavin measured by qRT-PCR. *indicate statistically significant differences between ROS25 and WT samples (*p*-value < 0.05).

In order to assess the effect of the point mutation in the FMN riboswitch region of ROS25, the transcription level of *ribD* (i.e., the first gene of the *rib* operon) was compared between the WT and strain ROS25 during the mid-exponential growth phase in mSM7lac (Figure 3). When riboflavin was not available in the medium, transcription of *ribD* was 15-fold higher in ROS25 than in WT (*p*-value < 0.05). Addition of 20 ng mL^–1^ riboflavin to the culture resulted in reduced *ribD* transcription in WT (*p*-value < 0.05), but did not exert a feedback repressive effect in ROS25, where the response of the riboswitch was lost and the *rib* operon was shown to be constitutively expressed.

To assess the global transcriptional effect of the mutation in the riboswitch that caused riboflavin overproduction in ROS25, an RNA-seq experiment was performed on WT and ROS25 cultures growing in mSM7lac without riboflavin supplementation (Supplementary Table S1). The genes of the *rib* cluster were the most differentially expressed between WT and ROS25. Transcription levels of this cluster in ROS25 were 18-fold higher than its counterpart in WT. Furthermore, various ABC transporters and sugar permeases were highly expressed in ROS25, suggesting a more active sugar metabolism in the mutant than in WT. Genes encoding glycosyltransferases involved in cell wall metabolism and extracellular polysaccharide biosynthesis were also upregulated in ROS25 (Supplementary Table S1). Besides the point mutation in the FMN riboswitch, additional genetic changes may be present in the genome of ROS25, which may explain the above transcriptional differences. Transcription of *ribC* was similar in both strains, while the genes encoding FAD-dependent proteins appear to be downregulated in ROS25 (*p*-value < 0.05; Supplementary Table S1).

### Effect of Carbon Sources on Riboflavin Production

**FIGURE 4:**
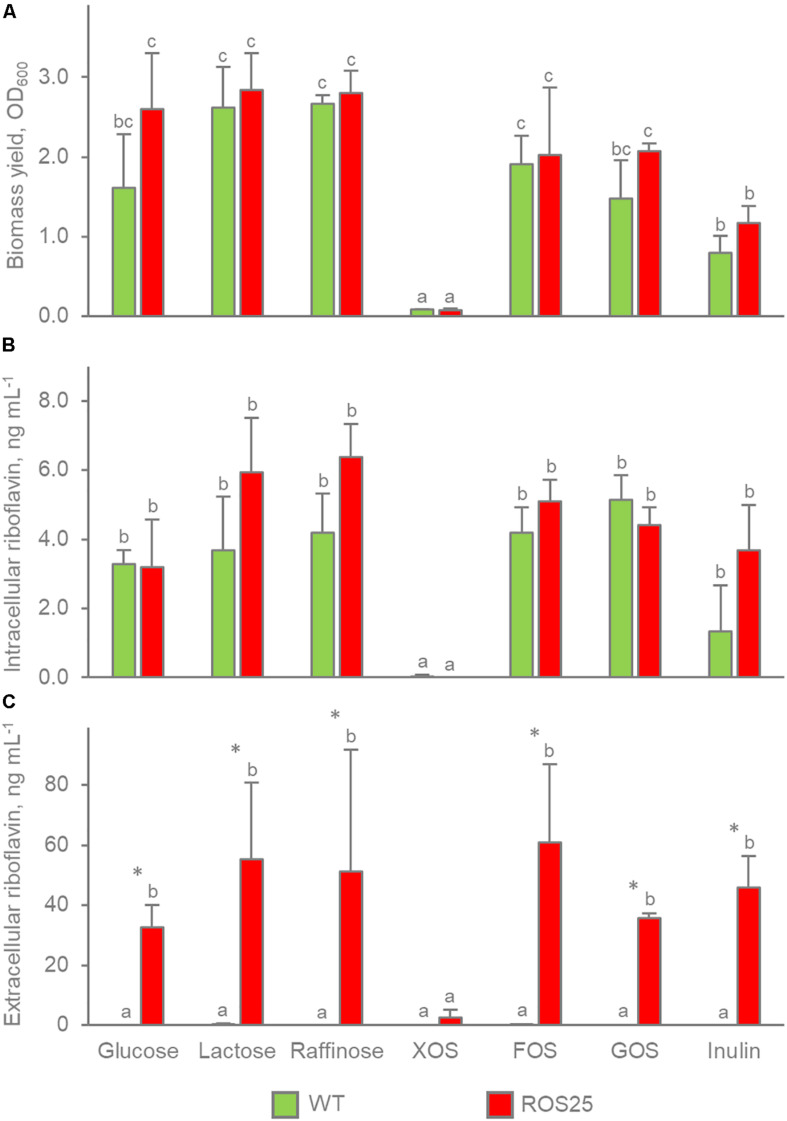
Growth yield (OD_600nm_) and riboflavin production in batch cultures of ROS25 and wild-type (WT) grown in mSM7 media containing varying carbohydrate sources (1%). **(A)** OD_600nm_; **(B)** intracellular riboflavin ng mL^–1^ of culture; **(C)** extracellular riboflavin ng mL^–1^ of supernatant. Values are means ± SD, *n* = 3. * indicates statistically significant differences between ROS25 and WT samples (*P* < 0.05); lowercase letters indicate statistical significant differences for the same strains on the diverse carbon sources between (*p*-value < 0.05).

To assess the effect of different carbon sources on riboflavin production, the intracellular and extracellular production of the vitamin was determined in batch cultures of WT and ROS25 grown in mSM7 containing glucose, lactose, raffinose, GOS, XOS, FOS or inulin as the sole carbon source. Both WT and ROS25 grew well with all the carbon sources except XOS (Figure 4). The growth yield was similar with glucose, lactose, raffinose, FOS, and GOS (*P* > 0.05), but lower with inulin (*p*-value < 0.05). Notably, the lowest biomass yield was obtained on inulin, presumably due to the presence of long chains that are not metabolized by *B. longum* subsp. *infantis*, as reported previously (Kim et al., 2013).

Except for XOS, where poor growth resulted in negligible amounts of vitamin B_2_ production, the carbon source did not affect the concentration of riboflavin accumulated within the cells or released in the supernatant by both WT and ROS25 cultures (*p*-value > 0.05). WT and ROS25 presented a similar concentration of intracellular riboflavin regardless of the carbon source (*p*-value > 0.05). In contrast, the difference between WT and ROS25 was significant with respect to the concentration of vitamin released in the medium (*p*-value < 0.05). The supernatants obtained from WT cultures typically contained < 0.2 ng mL^–1^ riboflavin, while those corresponding to ROS25 reached up to 60.8 ng mL^–1^ for a culture grown on FOS as the carbon source.

### Riboflavin Production and Release by ROS25

**FIGURE 5:**
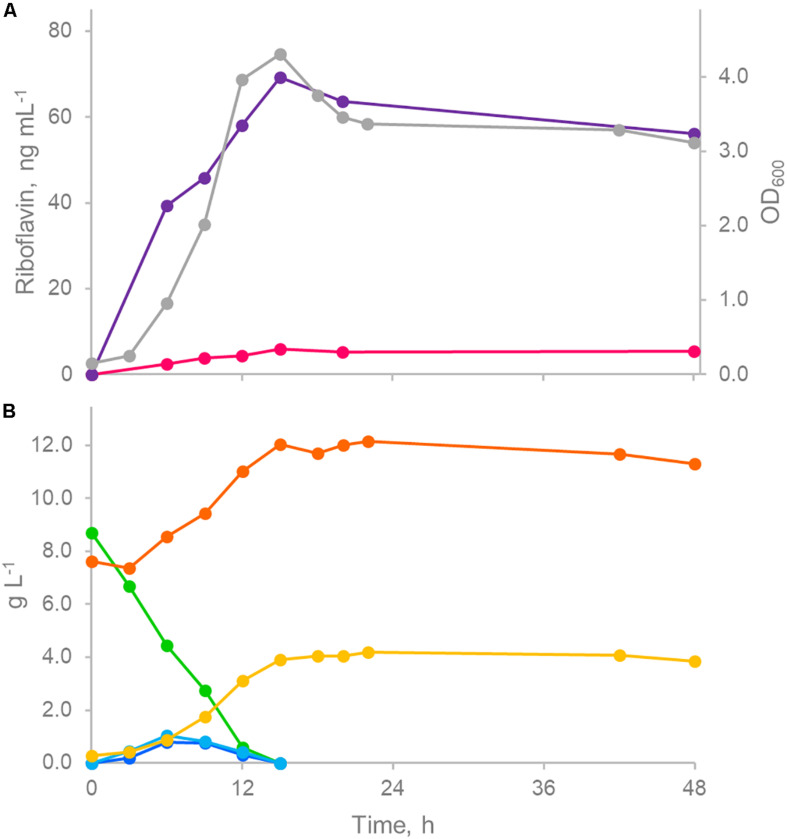
Controlled pH batch fermentation of ROS25 in mSM7lac. **(A)** Time course of biomass (gray) and riboflavin production (intracellular, fuchsia; extracellular, purple). **(B)** Time course of sugars (lactose, green; galactose, light blue; glucose, blue) and fermentation products (acetic acid, orange; lactic acid, green). The figure represents one representative experiment of the triplicate.

The kinetics of growth and associated riboflavin production was studied during pH-controlled batch fermentations of ROS25 in mSM7lac (Figure 5). Following a short lag phase, the culture rapidly grew and entered the stationary phase after 15 h, when lactose and the monosaccharides generated by its hydrolysis got exhausted. Accumulation of acetic and lactic acids originating from carbohydrate fermentation was observed concomitant with growth (Figure 5), while riboflavin was released in the medium throughout growth as well, reaching the highest concentration (65.3 μg L^–1^) when the culture entered into the stationary phase, then slightly declined. The highest rate of vitamin production (13.9 ng mL^–1^ h^–1^) was observed after 6 h of growth when the specific growth rate was also at its highest level (0.31 h^–1^).

**FIGURE 6:**
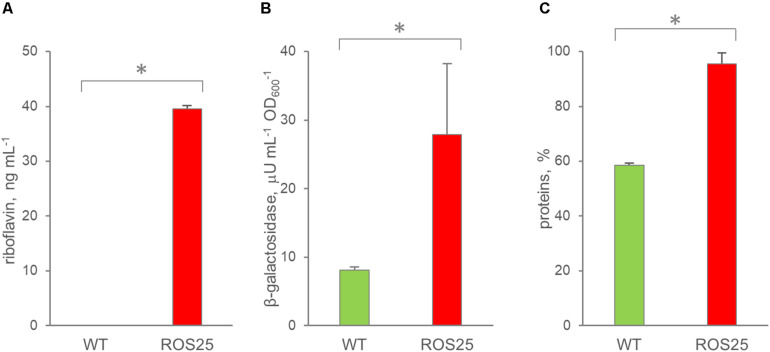
Permeability of wild-type (WT) and ROS25 cultures grown for 6 h in mSM7lac. Dosage of **(A)** extracellular riboflavin; **(B)** β-galactosidase, **(C)** proteins in the supernatant. Values are means ± SD, *n* = 3. * indicates statistically significant differences between ROS25 and WT samples (*p*-value < 0.05).

The extracellular concentrations of riboflavin, β-galactosidase, and total protein content were compared with those obtained for WT in order to assess if riboflavin was released in the supernatant by ROS25 due to cell lysis. After 6 h of growth in mSM7lac, WT and ROS25 cultures presented the same turbidity, but the latter had a significantly higher level of extracellular riboflavin, β-galactosidase, and total protein content (Figure 6). These data indicate higher cell wall permeability or partial lysis of ROS25 compared to a similarly grown WT.

### Production of Riboflavin in Fecal Cultures Supplemented With WT and ROS25

To assess whether consumption of ROS25 as probiotic would result in *in situ* riboflavin production which could benefit the host, the mutant was employed in fecal fermentations. Fresh feces of 10 healthy adults presented a riboflavin concentration ranging between 80.7 and 728.2 ng g^–1^, with a mean of 298.4 and a median of 251.5 ng g^–1^ (Supplementary Figure S3). The six samples with the lowest concentration were selected to prepare fecal cultures and inoculated (1:100 w/v) in mSM7GOS medium. Changes in riboflavin concentration were determined following inoculation with 10^6^ cfu mL^–1^ of ROS25, when compared to inoculation of the same level of WT and to control cultures that were not supplemented with bifidobacteria. To discriminate between riboflavin originating from the bifidobacterial supplement and that from other fecal bacteria, a parallel set of fermentations was carried out, where the fecal inoculum was heat treated prior to bifidobacterial inoculation.

**FIGURE 7:**
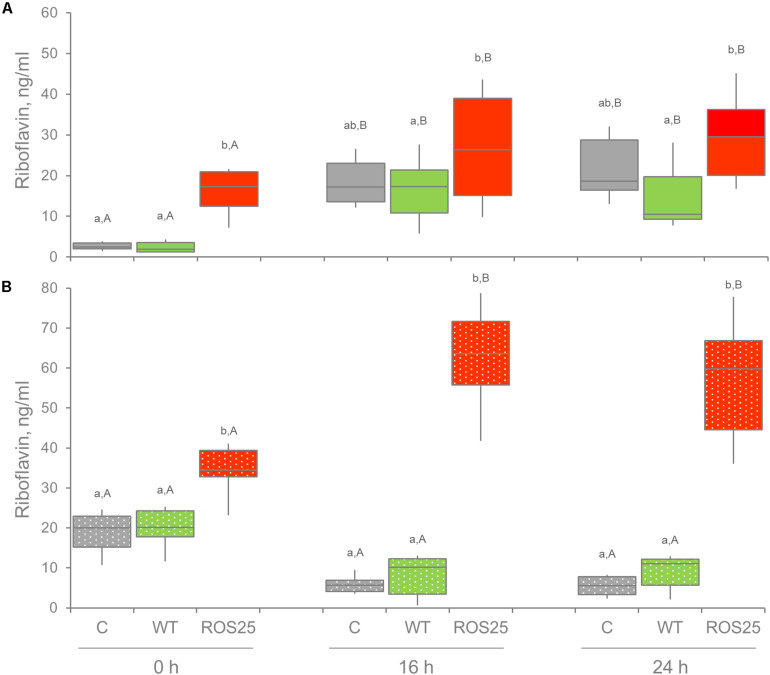
Extracellular riboflavin in fecal cultures. Riboflavin concentration was determined in the supernatant of fecal cultures at 0, 16, and 24 h of incubation in the absence of supplements (C) and in presence of riboflavin producing bifidobacteria [wild-type (WT) and ROS25]. **(A)** Fecal cultures with alive microbiota; **(B)** fecal cultures with pasteurized gut microbiota. Lower letters indicate statistically significant differences at the same time point among C, WT, and ROS25 cultures (*p*-value < 0.05); upper case letters indicate statistically significant differences for the same culture at various time points (*p*-value < 0.05).

With each fecal sample, heat treatment of feces and subsequent supplementation with ROS25 yielded the biggest change in riboflavin concentration after 16 or 24 h of incubation (Figure 7B), with a mean value of 61.2 ng mL^–1^. Under the same conditions, the cultures inoculated with WT yielded a mean of 7.9 ng mL^–1^, significantly lower than ROS25 and similar to the control.

All cultures inoculated with viable fecal bacteria accumulated riboflavin during the first 16 h of the process (*p*-value < 0.05), irrespective of bifidobacterial supplementation. This indicated that other members of microbiota are producing riboflavin as well. Following 16 and 24 h of incubation, riboflavin concentration was the highest in the cultures supplemented with ROS25 and the lowest in those supplemented with WT (*p*-value < 0.05). Nonetheless, values were widely dispersed within groups, thus the difference between ROS25, WT and the control groups was not significant.

## Discussion

Bifidobacteria are widely used as probiotics for their recognized health benefits and the ability to synthesize important biomolecules, e.g., vitamins, exopolysaccharides, short-chain fatty acids, etc. (Bottacini et al., 2017). The *de novo* synthesis of riboflavin represents a relatively uncommon feature in bifidobacterial genomes, being mainly found in representative species isolated from primates and just in one human gut commensal, *B. infantis*. Presumably, riboflavin synthesis is not crucial for survival of auxotrophic bifidobacterial species as other members of gut microbiota release this vitamin (Sharma et al., 2019). Even if the host diet does not supply a sufficient amount of riboflavin for every member of the gut microbiome, vitamin sharing allows successful colonization of the gut by bifidobacteria. For this reason, riboflavin overproducers holding probiotic attributes could be potential candidates for *in situ* production of the vitamin, once these strains reach the host intestine (Arena et al., 2014). Considering the extensive application of bifidobacteria in the food and pharmaceutical fields, coupled with consumer demand for healthier foods, the use of food-grade microorganisms as *in situ* vitamin delivery systems represents an attractive alternative to food fortification (LeBlanc et al., 2013, 2020; Steinert et al., 2016).

In the current study we performed an extensive comparative genome analysis of members of the genus *Bifidobacterium*, which allowed the identification of the genes required for uptake and metabolism of riboflavin (*ribU* and *ribCF*), both of which represent housekeeping and essential gene functions. A *de novo* biosynthetic pathway of this vitamin was previously predicted to be present in *B. infantis*, however, our comparative study identified for the first time a complete *rib* cluster (*ribDEABH*) in several bifidobacterial representative taxa, among which *B. infantis* is the only human commensal, thus making this species particularly relevant for the development of probiotic riboflavin overproducers. Our comparative genomics and phylogenomic analysis suggests that the acquisition of the *rib* cluster by *B. infantis* was the result of a horizontal transfer event, which is different from that observed in other non-human residential bifidobacteria, and likely originating from *Streptococcus* or *Clostridium*.

It is worth mentioning that *B. infantis* represents an unusual bifidobacterial strain with a “selfish” strategy of gut colonization characterized by efficient consumption of available carbohydrates (e.g., Human Milk Oligosaccharides structures or HMOs), thus making the saccharolytic metabolism of this species particularly demanding in terms of redox cofactors (e.g., FAD). For this reason, the acquisition of the *rib* cluster by *B. infantis* may have represented an important additional supply of riboflavin and FAD in support of its metabolism and strategy of gut colonization. *B. infantis* was specifically isolated from an infant gut, thus riboflavin synthesis could be a specific adaptation to the infant gut or diet (Sela et al., 2008).

The toxic riboflavin analog roseoflavin was used to isolate spontaneous roseoflavin-resistant mutants that upon further analysis exhibit riboflavin overexpression. All mutations that had occurred in the *rib* operon promoter were mapped in conserved regions of the predicted FMN riboswitch sequence located in the promoter region of the riboflavin biosynthesis cluster. Analysis of the spontaneous mutant ROS25, which was to shown to exhibit the highest production of riboflavin among the assessed mutants, indicated that a G → A transition in the anti-antiterminator region had caused deregulation of the FMN riboswitch. As a result of this deregulation, the riboflavin biosynthesis became constitutive in *B. infantis* ROS25, with an excess of this vitamin diffusing into the growth medium. Curiously, no deletions or insertions were found in the FMN riboswitch region, which is in contrast to observations made for *L. lactis* and *L. plantarum* (Burgess et al., 2004; Ge et al., 2020). Transitions are the most frequent type of point mutations occurring in bacteria, being induced by spontaneous tautomeric shifts (i.e., transient changes to an alternative form of a nucleobase molecule) which lead to base mispairing during replication (Griffiths et al., 2000). Apparently, high riboflavin production does not pose a constricting metabolic burden on a cell when grown under favorable laboratory conditions.

Despite the fact that superior analytical methods can be employed to provide a more reliable riboflavin quantification (e.g., HPLC and fluorescence; Mohedano et al., 2019), our screenings based on an established microbiological assay clearly demonstrate riboflavin overproduction in ROS25 and other obtained roseoflavin-resistant derivatives when compared to the WT strain ATCC 15697).

The mechanism by which riboflavin was released by ROS25 remains unclear. Comparison of permeability of exponential-growth cultures of WT and ROS25 indicated a more permeable cell envelope in ROS25 when compared to WT that, however, did not affect biomass yields and robustness of the culture. The mechanism underlying augmented permeability deserves deeper investigation, as it was not evident from RNAseq analysis.

Even though fecal cultures are artifacts that do not reflect the *in vivo* situation, where many factors affect the balance between vitamin production and consumption, including absorption by the host, the vitamin overproducing derivative ROS25 increased riboflavin level in both pasteurized and unpasteurized fecal cultures. Thus, this strain deserves to be challenged in a study *in vivo*, to find whether it can be successfully utilized as a probiotic vitamin “factory” improving the vitamin status of the host. Since no genetic engineering techniques were used to modify this strain, ROS25 and similar strains could be readily used to replace the standard probiotic strain in food formulations and would supply their host with the required vitamin intake. According to our results, the strain increased riboflavin level in both pasteurized and unpasteurized fecal cultures. Sharing of B-group vitamins has been suggested to promote stability in gut microbial communities (Sharma et al., 2019).

Besides the direct benefit to the host, such riboflavin production in the gut could help to stabilize bifidobacterial species that are auxotrophic for this vitamin. In fact, recent studies have explored the possibility of using vitamins (e.g., riboflavin, niacin) as prebiotics in order to stimulate a selected population of beneficial gut microbiota (Steinert et al., 2016; Fangmann et al., 2018).

## Data Availability Statement

The datasets presented in this study can be found in online repositories. The names of the repository/repositories and accession number(s) can be found in the article/Supplementary Material. RNA sequencing raw data are available from the Sequence Read Archive (SRA) database under the SRA accession PRJNA639329.

## Author Contributions

FB and DvS conceived the study. FB, AS, AA, SR, and MR designed the experiments. EE, AS, AA, and SR carried out the experiments. FB, AS, DvS, AA, SR, and MR analyzed the data. FB, AS, DvS, AA, SR, and MR wrote the manuscript. All authors discussed the results and commented on the manuscript.

## Conflict of Interest

The authors declare that the research was conducted in the absence of any commercial or financial relationships that could be construed as a potential conflict of interest.

## Footnotes

1 ftp://ftp.ncbi.nlm.nih.gov/genomes/refseq/bacteria

2 https://www.r-project.org/about.html

3 https://github.com/bioconda

4 https://alignmentviewer.org

5 https://bacteria.ensembl.org

6 http://bowtie-bio.sourceforge.net/bowtie2/index.shtml
