# Supplementary material for: Riboflavin Biosynthesis and Overproduction by a Derivative of the Human Gut Commensal Bifidobacterium longum subsp. infantis ATCC 15697
Source: Front Microbiol. 2020 Sep 15;11:573335. doi: 10.3389/fmicb.2020.573335 (PMC7522473; doi:10.3389/fmicb.2020.573335)
Supplement: Supplementary file 1 [file Table_1.docx]

Supplementary Material

# Supplementary Figures and Tables

## Supplementary Figures


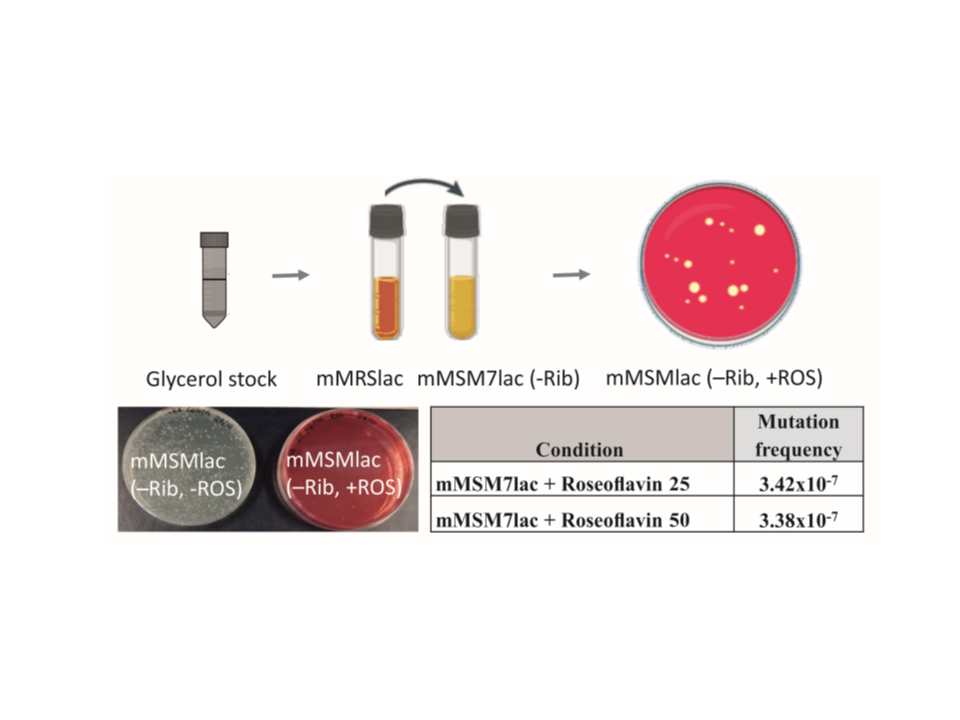


**Supplementary Figure 1. Isolation of roseoflavin resistant mutants.** Isolation of roseoflavin-resistant isolates. 5 ml WT overnight culture in mMRSlac (modified MRS medium supplemented with 1 % lactose) was washed and re-suspended in 10 ml of mSM7lac (modified SM7 medium supplemented with 1 % lactose). The culture was allowed to grow for 5 hours under anaerobic conditions. Aliquots of 100 μl of cell suspension were spread-plated on solidified media containing various concentrations of roseoflavin (0, 25 or 50 μg ml-1).


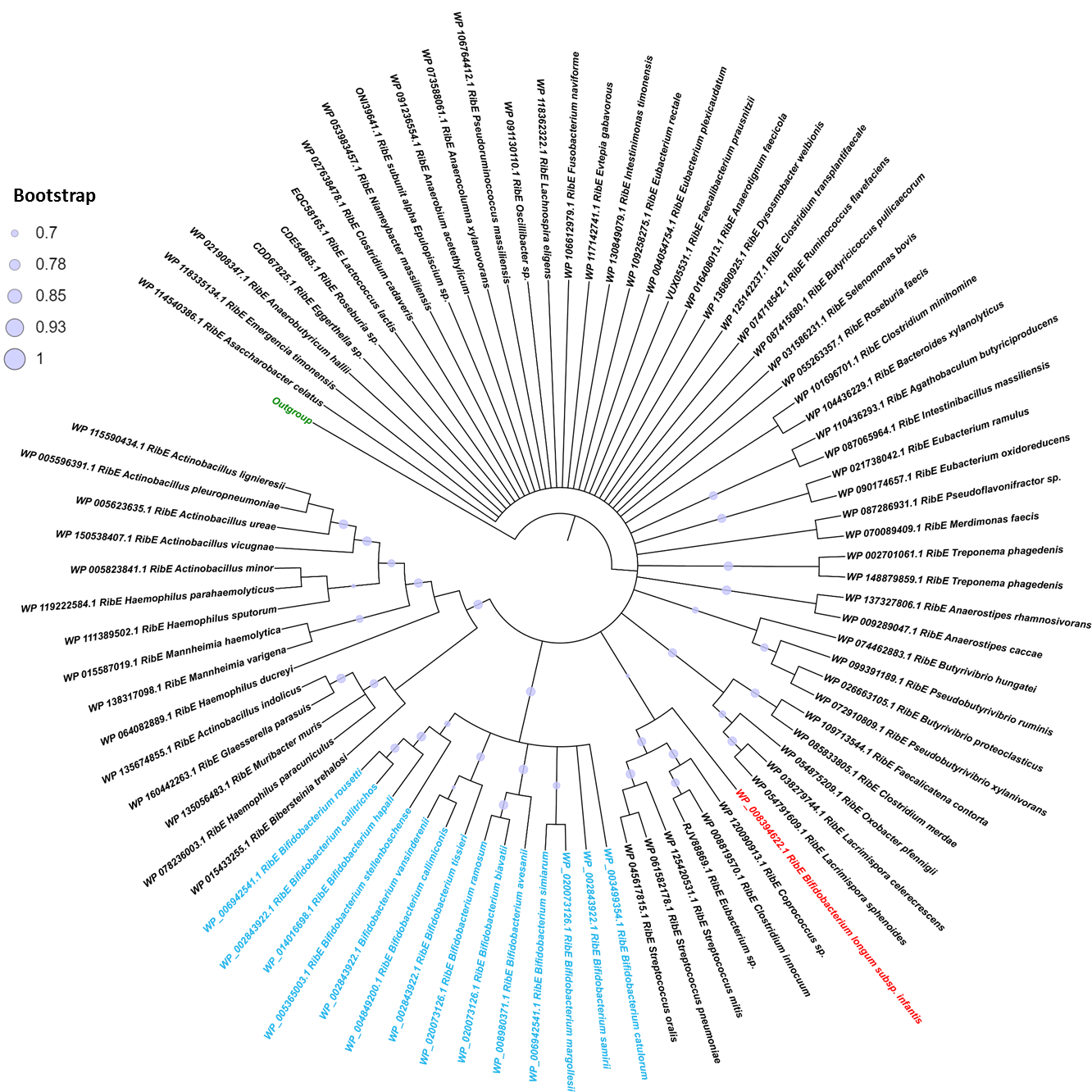


**Supplementary Figure 2.** **The *rib* cluster in gut bacteria.** Neighbour Joining tree showing the phylogenetic relationship of homologous of the *ribE* gene in 84 gut bacteria as identified by PSI-BLAST alignment. The NJ tree was generated using the MEGA suite and a statistical validation of 100 bootrap replicates. The tree was visualised using iTOL (https://itol.embl.de). Highlighted in red is the *ribE* gene identified in *B. longum* subsp. *infantis* ATCC15697 and in pale blue are highlighted the homologous *ribE* genes identified in other bifidobacteria. From the obtained tree is possible to observe that the *ribE* in ATCC15697 sits in a separate clade together with other human gut bacteria.


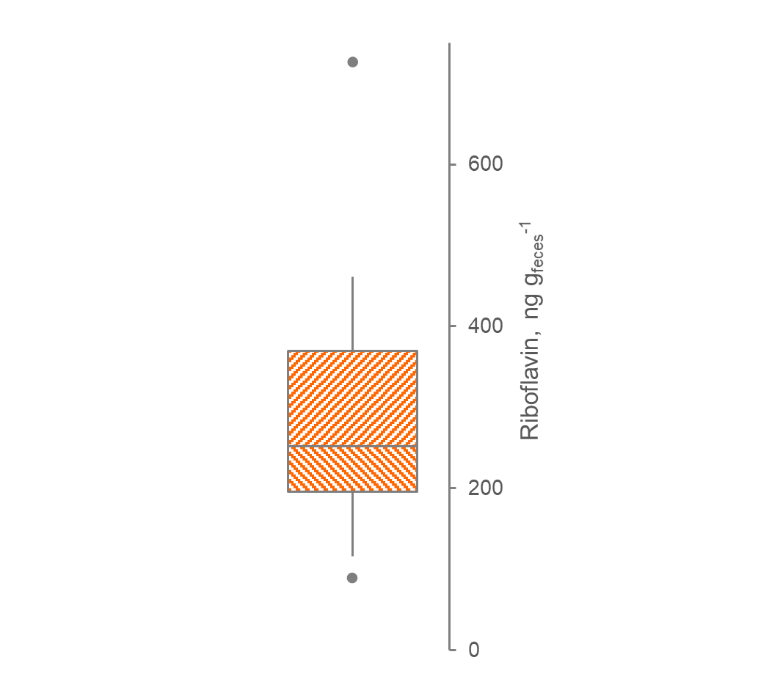


**Supplementary Figure 3. Riboflavin content in fresh feces.** The boxplot represents the content of riboflavin in fresh feces of 10 healthy adults. Box indicate the median and 25th and 75th percentiles; whiskers indicate 10th and 90th percentiles; dots represents the highest and the lowest values.

# Supplementary Tables

## Supplementary Table 1. Significant (p<0.05) differentially expressed genes in ROS25 vs WT, grown in mSM7lac (-Rib), as determined by RNA sequencing.

| Gene | Fold expression ROS25 vs WT | Annotation |
| --- | --- | --- |
| Blon_0388 | 20.8 | GTP cyclohydrolase II RibAB |
| Blon_0386 | 17.6 | riboflavin biosynthesis protein RibD |
| Blon_0389 | 17.1 | 6,7-dimethyl-8-ribityllumazine synthase RibH |
| Blon_0387 | 16.7 | riboflavin synthase alpha subunit RibE |
| Blon_1540 | 19.8 | hypothetical protein |
| Blon_1027 | 13.9 | hypothetical protein |
| Blon_1028 | 13.1 | hypothetical protein |
| Blon_1782 | 12.9 | protein of unknown function DUF450 |
| Blon_1026 | 12.4 | hypothetical protein |
| Blon_1536 | 12.1 | hypothetical protein |
| Blon_1541 | 12.0 | hypothetical protein |
| Blon_2342 | 10.8 | binding-protein-dependent transport systems inner membrane component |
| Blon_2325 | 10.8 | 3-isopropylmalate dehydratase large subunit |
| Blon_2343 | 10.8 | binding-protein-dependent transport systems inner membrane component |
| Blon_0996 | 10.7 | conserved hypothetical protein |
| Blon_2345 | 10.5 | binding-protein-dependent transport systems inner membrane component |
| Blon_2346 | 10.5 | binding-protein-dependent transport systems inner membrane component |
| Blon_0390 | 10.3 | Endonuclease/exonuclease/phosphatase |
| Blon_0594 | 10.3 | citrate synthase I |
| Blon_0290 | 10.0 | Glyoxalase/bleomycin resistance protein/dioxygenase |
| Blon_0453 | 9.8 | hypothetical protein |
| Blon_1783 | 9.8 | conserved hypothetical protein |
| Blon_2160 | 9.7 | 1 4-dihydroxy-2-naphthoate octaprenyltransferase |
| Blon_1534 | 9.7 | hypothetical protein |
| Blon_2106 | 9.6 | glycosyl transferase family 2 |
| Blon_0606 | 9.5 | nucleoside 2-deoxyribosyltransferase, nucleobase recycling |
| Blon_1539 | 9.5 | hypothetical protein |
| Blon_0526 | 9.4 | helix-turn-helix domain protein |
| Blon_1371 | 9.2 | tryptophan synthase beta subunit |
| Blon_2107 | 9.1 | glycosyl transferase group 1 |
| Blon_0521 | 9.0 | Lanthionine synthetase C family protein |
| Blon_1538 | 8.9 | addiction module antitoxin RelB/DinJ family |
| Blon_2093 | 8.8 | hypothetical protein |
| Blon_0984 | 8.8 | hypothetical protein |
| Blon_1495 | 8.6 | conserved hypothetical protein |
| Blon_0520 | 8.5 | hypothetical protein |
| Blon_0525 | 8.4 | hypothetical protein |
| Blon_0995 | 8.3 | ABC transporter related |
| Blon_2355 | 8.3 | glycoside hydrolase family 20 |
| Blon_2108 | 8.2 | hypothetical protein |
| Blon_2109 | 8.1 | glycosyl transferase family 2 |
| Blon_0244 | 8.1 | Signal transduction histidine kinase-like protein |
| Blon_1735 | 8.0 | conserved hypothetical protein |
| Blon_0475 | 7.7 | ABC transporter related |
| Blon_2354 | 7.7 | extracellular solute-binding protein family 1 |
| Blon_2386 | 7.6 | ABC transporter related |
| Blon_1781 | 7.6 | filamentation induced by cAMP protein Fic |
| Blon_0009 | 7.6 | ATPase |
| Blon_0581 | 7.6 | ABC transporter related |
| Blon_0131 | 7.6 | ketol-acid reductoisomerase |
| Blon_0011 | 7.5 | Glutamate dehydrogenase NADP |
| Blon_0448 | 7.4 | hypothetical protein |
| Blon_2452 | 7.4 | threonine dehydratase |
| Blon_2177 | 7.4 | extracellular solute-binding protein family 1 |
| Blon_0631 | 7.4 | membrane lipoprotein lipid attachment site |
| Blon_0518 | 7.3 | hypothetical protein |
| Blon_2351 | 7.3 | extracellular solute-binding protein family 1 |
| Blon_0884 | 7.3 | binding-protein-dependent transport systems inner membrane component |
| Blon_0517 | 7.1 | ABC transporter related |
| Blon_2110 | 7.1 | glycosyltransferase |
| Blon_2385 | 7.1 | glycosyl transferase family 8 |
| Blon_0285 | 7.1 | alpha/beta superfamily hydrolase |
| Blon_0519 | 7.1 | hypothetical protein |
| Blon_1577 | 7.0 | NAD P transhydrogenase beta subunit |
| Blon_2070 | 7.0 | 3-isopropylmalate dehydrogenase |
| Blon_1685 | 7.0 | ABC transporter related |
| Blon_0850 | 7.0 | Peptidoglycan glycosyltransferase |
| Blon_2350 | 6.8 | extracellular solute-binding protein family 1 |
| Blon_1854 | 6.7 | ABC transporter related |
| Blon_0826 | 6.7 | conserved hypothetical protein |
| Blon_1259 | 6.6 | Allergen V5/Tpx-1 family protein |
| Blon_0166 | 6.6 | aspartate-semialdehyde dehydrogenase |
| Blon_2384 | 6.5 | hypothetical protein |
| Blon_2387 | 6.5 | ABC-2 type transporter |
| Blon_0028 | 6.4 | conserved hypothetical protein |
| Blon_1146 | 6.4 | conserved hypothetical protein |
| Blon_1494 | 6.4 | hypothetical protein |
| Blon_0883 | 6.4 | extracellular solute-binding protein family 1 |
| Blon_0819 | 6.3 | 67 kDa myosin-cross-reactive antigen family protein |
| Blon_2114 | 6.2 | Undecaprenyl-phosphate galactose phosphotransferase |
| Blon_0204 | 6.2 | conserved hypothetical membrane protein |
| Blon_1362 | 6.2 | protein of unknown function DUF881 |
| Blon_0164 | 6.1 | aspartate kinase |
| Blon_0845 | 6.0 | D-isomer specific 2-hydroxyacid dehydrogenase NAD-binding |
| Blon_2352 | 5.5 | extracellular solute-binding protein family 1 |
| Blon_2054 | 5.3 | protein of unknown function DUF711 |
| Blon_2392 | 5.0 | conserved hypothetical protein |
| Blon_1050 | 4.7 | conserved hypothetical protein |
| Blon_0914 | -5.9 | GTP-binding protein TypA |
| Blon_1696 | -6.4 | O-acetylhomoserine/O-acetylserine sulfhydrylase |
| Blon_2016 | -7.2 | Beta-galactosidase |
| Blon_2183 | -7.6 | PTS system glucose subfamily IIA subunit |
| Blon_0178 | -8.1 | phosphoenolpyruvate-protein phosphotransferase |
| Blon_0102 | -8.2 | peptidase S1 and S6 chymotrypsin/Hap |
| Blon_0035 | -9.1 | alkyl hydroperoxide reductase/ Thiol specific antioxidant/ Mal allergen |
| Blon_1963 | -9.5 | periplasmic solute binding protein |
| Blon_2444 | -9.9 | extracellular solute-binding protein family 1 |
| Blon_2200 | -13.4 | conserved hypothetical protein |
| Blon_2035 | -14.2 | Dihydrodipicolinate reductase (lysine biosynthesis) |
| Blon_1633 | -22.6 | two component transcriptional regulator LuxR family |
| Blon_0833 | -22.7 | binding-protein-dependent transport systems inner membrane component |
| Blon_1019 | -22.8 | putative metalloendopeptidase glycoprotease family |
| Blon_1081 | -27.0 | conserved hypothetical myosin-like protein with unknown function |
| Blon_0834 | -28.1 | extracellular solute-binding protein family 5 |
